# The direction of carbon and nitrogen fluxes between ramets in *Agrostis stolonifera* changes during ontogeny under simulated competition for light

**DOI:** 10.1093/jxb/ery068

**Published:** 2018-02-21

**Authors:** Jana Duchoslavová, Jan Jansa

**Affiliations:** 1 Department of Botany, Faculty of Science, Charles University, Benátská, Czech Republic; 2 Institute of Microbiology, Czech Academy of Sciences, Vídeňská, Czech Republic

**Keywords:** Carbon, clonal plant, development, light, nitrogen, ontogeny, physiological integration, ramet, stable isotopes, translocation

## Abstract

Resource sharing is universal among connected ramets of clonal plants and is driven both by the developmental status of the ramets and the resource gradients. Above-ground competition forms spatial light gradients, but the role of resource sharing in such competition is unclear. We examined translocation of resources between mother and daughter ramets of *Agrostis stolonifera* under light heterogeneity throughout ramet ontogeny. We labelled ramets with ^13^C and ^15^N to estimate the bidirectional translocation of resources at three developmental stages of the daughters. In addition, we compared the final biomass of integrated and severed ramets in order to estimate the effect of integration on growth. Young developing daughters were supported by carbon, whereas nitrogen was only translocated towards daughters at the beginning of rooting, regardless of the light conditions. Shading of mothers was a major determinant of resource translocation between developed ramets, with carbon being preferentially moved to daughters from shaded mothers while nitrogen translocation was limited from daughters to shaded mothers. Surprisingly, the absolute amounts of translocated resources did not decline during development. Growth of daughters was enhanced by integration regardless of the shading. Overall, *A. stolonifera* maximizes the resource translocation pattern in order to enable it to spread from unfavourable habitats, rather than compensating for light heterogeneity among ramets.

## Teaser

The dynamics of carbon and nitrogen translocation between mother and daughter ramets of a clonal grass change during ramet ontogeny and are influenced by the light conditions experienced by the mother ramets.

## Introduction

Clonal plants can form multiple ramets (i.e. potentially independent units with their own shoots and roots), which are interconnected by stolons or rhizomes. The connections between ramets enable plants to share resources and information through the integrated system (Marshall, 1990; Song *et al.*, 2013), which may provide them with an advantage over non-integrated plants that rely solely on the resources present in their patch, especially when resources are distributed heterogeneously (e.g. Friedman and Alpert, 1991). Resource translocation is particularly important for new, developing ramets, which are supported by water, mineral nutrients, and/or photosynthates from older and more developed ramets (Hartnett and Bazzaz, 1983; Pitelka and Ashmun, 1985; Marshall, 1990). Due to the parental support, new ramets can have an advantage over seedlings when they are growing in competitive or stressful conditions (Sarukhan and Harper, 1973). However, it has also been shown that mineral nutrients may be preferentially translocated from daughter to larger parental ramets to meet the higher nutrient demand of the latter (Pinno and Wilson, 2014). The initial resource translocation may decline as ramets develop and become self-sustainable in resource acquisition (Hartnett and Bazzaz, 1983). However, exchange of resources can be maintained even among developed ramets, and it can be induced or enhanced by heterogeneity in resource availability, local stress, or differential resource needs of the ramets (Pitelka and Ashmun, 1985; Song *et al.*, 2013). Accordingly, benefits of clonal integration have been shown to increase with increasing heterogeneity in the availability of water (Pennings and Callaway, 2000), mineral nutrients (Alpert, 1991, 1996; Birch and Hutchings, 1994), or light (Stuefer *et al.*, 1994; Xu *et al.*, 2010).

Heterogeneity in both light and below-ground resources can be due to the abiotic environment or generated by plant interactions themselves (Chazdon and Pearcy, 1991; Skálová *et al.*, 1999). Vegetation may form strong gradients in light availability, especially when below-ground resources are abundant, and competition for light then becomes the dominant factor affecting plant growth. Therefore, the ability to cope with light heterogeneity may be essential for plants in such environments. In addition, smaller plants are disproportionally handicapped because competition for light is size-asymmetric (Weiner, 1990). Integration of ramets may allow clonal plants to cope with light heterogeneity by support of (temporarily) shaded ramets and thereby partially compensate for the asymmetry of light competition (de Kroon *et al.*, 1992). Indeed, benefits of clonal integration for shaded ramets have been demonstrated by growth experiments (e.g. Hartnett and Bazzaz, 1983; Stuefer *et al.*, 1994; Xu *et al.*, 2012), although in some cases, the parental support may cease when shading lasts too long (Hartnett and Bazzaz, 1983). In addition, the spread from open patches to neighbourhoods of other species is facilitated by clonal integration in some species (Roiloa *et al.*, 2010; Xiao *et al.*, 2011). In others, however, clonal integration mainly enhances the exploration of open space and the quick expansion into unvegetated patches (Pennings and Callaway, 2000). The effect of integration on the growth of the whole clonal plant under competition also differs among species (Peltzer, 2002; Pauliukonis and Gough, 2004; Wang *et al.*, 2016). The role of clonal integration in light competition is, therefore, still far from clear, although it has recently gained increasing attention, especially in connection with invasive species (e.g. Yu *et al.*, 2009; You *et al.*, 2014; Wang *et al.*, 2016).

Shading has been shown to affect carbon translocation between ramets in several plant species (Qureshi and Spanner, 1973; Pitelka and Ashmun, 1985). For example, in an experiment simulating clonal spread from a bare habitat (full sun) to vegetation shade (85% shade cloth), an enhanced transport of photosynthates from unshaded parent ramets to continuously shaded daughter ramets was observed in *Alternanthera philoxeroides*, whereas in *Phyla canescens* carbon import did not differ between shaded and unshaded daughter ramets (Xu *et al.*, 2010). Moreover, differences in growth of integrated and severed ramets of the two species corresponded to the observed differences in translocation (Xu *et al.*, 2010, 2012). In contrast to carbon, translocation of mineral nutrients in response to light gradients has to date been rarely studied, although differential availability of light may induce an imbalance in needs for nutrients in different ramets, even though nutrients in the substrate may be distributed homogeneously. Indeed, the results of Saitoh *et al.* (2006) indicated that nitrogen translocation from shaded to unshaded ramets could be enhanced due to higher sink activity of developing unshaded leaves. Moreover, the supply of shaded ramets with photosynthates can increase their ability to assimilate nitrogen from the soil (Chen *et al.*, 2015).

Patterns of resource translocation are likely to change markedly during ramet development. However, so far research has usually focused on a single resource type at a single point in time in ramet ontogeny (but see Alpert *et al.*, 2002; Luo *et al.*, 2014). Furthermore, the assessment of both the physiological and ecological relevance of nutrient flows is possible only if direct labelling of the specific resource is carried out so as to trace the transport in either direction. Combining the results of independent experiments is complicated by differing assimilation capacities and physiological status of experimental plants. Surprisingly, only a few studies have so far examined the transport of labelled resources in both directions between the parent and daughter ramets (but see Alpert, 1996; Pinno and Wilson, 2014). Thus, a coherent picture of net translocation of both carbon and nutrient resources among developing ramets in a heterogeneous environment is still lacking.

We addressed this gap by simultaneous examination of nitrogen and carbon flow between mother and daughter ramets during the development of the daughter ramet. We used pairs of mother and daughter ramets of a stoloniferous grass, *Agrostis stolonifera*, to study the exchange of carbon and nitrogen under heterogeneous light conditions, simulating above-ground competition through generating light gradients. We estimated bidirectional translocation of carbon and nitrogen between ramets at three developmental stages: at the very beginning of daughter rooting, during the initial daughter development, and finally when the size of the daughter reached that of the mother. At the same time, we measured the effect of clonal integration on plant growth under the same conditions.

We hypothesized that, in general, initial translocation of both resources towards daughters would decline with time as the daughters develop their own assimilation structures and become functionally independent of the mother. Furthermore, we expected a relatively strong effect of light gradient on the carbon flow between the ramets, with translocation directed preferentially to shaded daughter ramets, and reverse net carbon translocation from developed daughters to shaded mothers. With respect to nitrogen, we hypothesized that its translocation from shaded to unshaded ramets could be enhanced because of differential growth rates of shaded and unshaded ramets. In addition, daughter ramets with sufficiently developed roots may provide mother ramets with nitrogen from the newly occupied patch.

## Materials and methods

### Growth habit of *Agrostis stolonifera*

*Agrostis stolonifera* is a perennial stoloniferous grass, common in mesic and wet grasslands and river banks (Kik *et al.*, 1990; Kubát *et al.*, 2002). Clonal reproduction prevails over propagation through seeds in this species. Developed ramets are composed of multiple tillers, which may bend groundwards and develop into stolons, forming leaves and roots at some of their nodes. Connections among ramets persist for the whole vegetative season and can overwinter.

The plant material used in this experiment originated from a single genotype collected in a field and grown in an experimental garden since 2010. None of the plants flowered during the experiment.

### Initial cultivation

Tillers of source plants were cut and placed on wet sand to initiate rooting in mid-March 2016. Individual ramets (i.e. single nodes with developed leaves and roots) were then separated and planted in 1-l pots with a mixture of washed sand and slow-release fertilizer (3 g per pot, Substral Osmocote Grass, www.substral.cz; gravimetric nutrient content: N, 23%; P, 5%; K, 10%; Mg, 2%; S 9%) at the beginning of May 2016. These ramets are referred to as the mother ramets. The pots were positioned in a greenhouse equipped with supplemental lighting [400 W metal halide lamps providing a minimum of 200 μmol m^–2^ s^–1^ photosynthetically active radiation (PAR), extending the daylight period to 14 h] and watered three times a day with tap water. Dead ramets were replaced by new ones until the end of May 2016.

A shading cloth was installed 3 d before the establishment of daughter ramets. Plants were shaded from the top and all sides by the shading cloth combined with strips of green foil (3-cm wide, with 3-cm gaps in between, LeeFilters Fern Green 122, www.leefilters.com) to simulate changes in both light quantity and quality caused by above-ground competition (80% PAR reduction and 30% reduction of the red to far-red ratio). Shading also reduced the air temperature (see Supplementary Data Fig. S1 at *JXB* online for details).

### Experimental design

**Fig. 1.**
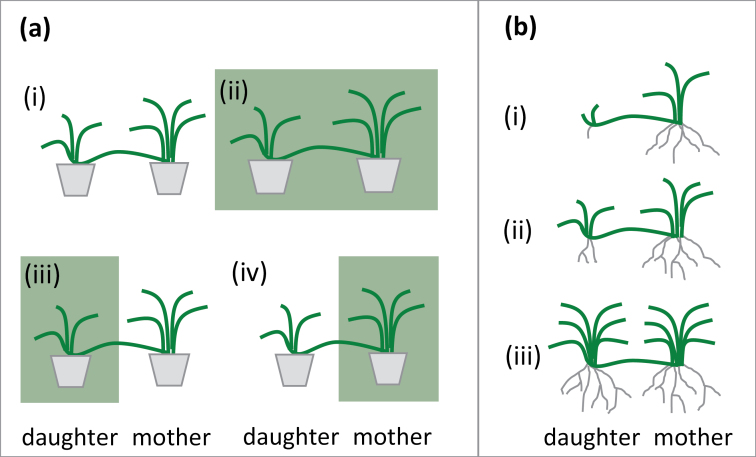
Design of the experiment. (a) Four shading treatments were used: (i) both ramets in full light, (ii) both ramets under green shade, (iii) daughter ramet shaded, or (iv) mother ramet shaded. (b) Schematic representation of ramet size at the time of harvests: (i) first (0 weeks), (ii) second (2 weeks), and (iii) final (8 weeks after daughter establishment).

Formation of daughter ramets was initiated over 3 d between 13–15 June 2016 by placing the longest stolon of each mother ramet in an adjacent vacant pot. For logistical reasons, each labelling and harvest campaign was also conducted over three subsequent days, so that the same intervals between the daughter ramet initialization and harvest were maintained for all plants for each age cohort of daughter ramets. Therefore, plant units (ramet pairs) processed at each harvest campaign (i.e. for a given ontogeny stage) were divided into three time-blocks with 1-d differences in initialization/harvest. Treatments were represented evenly among the blocks. A factorial design with mothers and daughters grown either in full light or under green shade was used (Fig. 1a).

Bidirectional translocation of carbon and nitrogen between mothers and daughters was examined by stable-isotope labelling (using ^13^C and ^15^N) at three developmental stages of the daughters: at the time of rooting (i.e. labelling took place immediately after daughter ramet initialization), 2 weeks after rooting, and 8 weeks after rooting (Fig. 1b). Four replicates (ramet pairs) were used for each combination of shading treatment, direction of translocation, and time of labelling.

### Stable-isotope labelling

Plants were pulse-labelled simultaneously with nitrogen (^15^N) and carbon (^13^C) according to a protocol tested in a pilot experiment (J. Duchoslavová and J. Jansa, unpublished results). Each labelling started at approximately 08.00 h (the photoperiod began at 06.00 h). Nitrogen was applied directly to pots by a syringe in the form of double-labelled ammonium nitrate (99 atom% ^15^N, 15 mg per pot). Carbon was applied in the form of ^13^CO_2_ (see Supplementary Data Figs S2, S3 for photographs), as follows. Labelled ramets (single pots) were enclosed in Plexiglass chambers equipped with a fan to mix the inner atmosphere, and ^13^CO_2_ was released inside the chambers by injecting 20 ml of phosphoric acid (20%, w:v) into a vial with ^13^C-enriched sodium carbonate (99% atom% ^13^C, 0.3 g per pot, calculated initial ^13^CO_2_ concentration inside chambers reaching 3100 ppm). The ramets were allowed to assimilate the labelled carbon for 2 h, and then the remaining CO_2_ in the atmosphere was scrubbed by circulating the air through NaOH solution (0.1 M, 200 ml) before opening the chamber. The labelling took place in full sunlight supplemented by additional light from diode lamps (LumiGrow Pro Series Pro 325 LED Lighting Systems, photosynthetic photon flux density 333 μmol m^–2^ s^–1^ at a distance of 1 m), and the plants were returned to the experimental shading conditions immediately after the labelling period. The mean light intensity inside the labelling chambers reached between 180–600 μmol m^–2^ s^–1^ PAR, depending on the day (data not shown).

Labelled ramet pairs were always harvested exactly 2 d after labelling. Mother and daughter ramets were separated; the roots were washed and separated from the shoots. The plant material was then dried (60 °C for 48 h), weighed, and milled to a fine powder using a ball mill (MM200, Retsch, Haan, Germany) before the elemental and isotopic analyses. The N and C concentrations and isotopic compositions of the two elements were measured using an elemental analyser (Flash EA 2000) coupled with an isotope-ratio mass-spectrometer (Delta V Advantage, ThermoFisher Scientific, Waltham, MA, USA).

To calculate the amount of ^13^C and ^15^N originating from pulse-labelling (i.e. excess ^13^C or ^15^N), *F*-ratios were calculated as *R*_S_/(*R*_S_ +1), where *R*_*S*_ is the molar isotope ratio in a sample (^15^N/^14^N or ^13^C/^12^C). The amount of total carbon or nitrogen (*C*, in moles) was then calculated as:

$$C=\left(W_{\text{dry}}\times B\right)/\left[\text{a}\times \left(F+\text{b}\right)\times \left(F–\text{1}\right)\right]$$ (1)

where *W*_dry_ is dry weight of a sample, *B* is molar concentration of carbon or nitrogen in a sample, a is 12 for carbon and 14 for nitrogen, b is 13 for carbon and 15 for nitrogen, and *F* is the *F*-ratio of the respective element in a sample. The amount of ^13^C and ^15^N originating from pulse-labelling (*E*, in moles) was finally calculated as:

$$E=\left(F_{\text{s}}–F_{\text{U}}\right)\times C$$ (2)

where *F*_*S*_ is the *F*-ratio of a sample and *F*_*U*_ is the mean *F*-ratio of four unlabelled control ramet pairs for each harvest. The amount of ^13^C and ^15^N originating from pulse-labelling that was found in unlabelled ramets (roots and shoots combined) is hereafter referred to as the amount of translocated ^13^C and ^15^N.

### Severed ramet pairs

To estimate the effect of integration on ramet growth (biomass), additional ramet pairs were cultivated in the same conditions as the plants used for isotopic labelling (with eight replicates per shading treatment). In these plants, the connections between ramets were severed 1 week after establishment of the daughter ramets. These additional plants were harvested at the time of the final harvest (i.e. 8 weeks after daughter ramet initialization) in the same way as the labelled plants, but they were not analysed for their elemental and isotopic composition.

### Data analyses

All statistical analyses were performed in the R statistical environment (version 3.3.2, www.r-project.org).

The effects of shading, time since daughter establishment, and direction of translocation on the amount of translocated label and on the proportion of the label exported from a labelled ramet were tested by separate linear models for carbon and nitrogen. Shading treatment was included in the models as shading of mothers and shading of daughters (i.e. two two-level factors). Time was included as a three-level factor, with the first harvest as a reference level. The proportion of exported label was calculated as the ratio of label amount in an unlabelled ramet and as the total label amount in a ramet pair. The proportion of exported labels as well as the absolute amount of translocated carbon and nitrogen were log-transformed to meet model assumptions. Effect estimates based on treatment contrasts and corresponding *t* and *P*_*t*_ values were used for interpretation of the models.

The effects of integration and shading on ramet growth were tested by separate linear models for mother and daughter ramets. The initial size of ramets, measured as total length of their tillers at the time of daughter establishment, was included as a covariate. The dry biomasses of integrated and severed ramets at the time of the final harvest were used as response variables, and they were log-transformed to meet model assumptions.

## Results

### Ramet size

At the time of the first harvest, mothers had on average 2.4 times higher dry biomass than daughters (Table 1), and the single, emerging roots of daughters were on average 7 mm long. At the time of the second harvest, mother ramets in full-sun and full-shade treatments had 2.1 times higher dry biomass than daughters (Table 1). When only daughters were shaded, mothers had 2.8 times higher dry biomass, and biomass of both ramets was similar when only mothers were shaded (Table 1). At the final harvest, the biomass of daughters in homogeneous treatments reached that of the mothers. When only one ramet was shaded, the ramets in the sun had 4.4–4.5 times higher biomass than the shaded ramets (Table 1).

**Table 1.** Biomass of daughter and mother ramets at the times of the first, second and final harvest

	Biomass of daughters (g)		Biomass of mothers (g)	
	Mean	SE	Mean	SE
First harvest*	0.07	0.01	0.17	0.02
**Second harvest**				
Full light	0.47	0.07	1.01	0.12
Daughter shaded	0.32	0.05	0.65	0.14
Mother shaded	0.17	0.02	0.16	0.03
Full shade	0.15	0.03	0.32	0.08
**Final harvest**				
Full light	6.91	0.53	6.07	0.52
Daughter shaded	1.12	0.18	5.11	0.74
Mother shaded	5.7	0.54	1.28	0.24
Full shade	0.35	0.06	0.43	0.09

*Biomass was not affected by the shading treatment due to its short duration at the first harvest.

### Translocation of carbon

The amount of translocated carbon was significantly affected by the direction of translocation and shading of mother ramets. The effects of direction and shading changed significantly with time. Shading of daughters did not have a significant effect on carbon translocation (for *F*- and *P*-values see Table 2).

**Table 2.** ANOVA of linear models of the amount of ^13^C and ^15^N (log-transformed) translocated towards unlabelled ramets in response to direction of translocation, shading of mothers (M) and daughters (D), and time of labelling

	d.f.	Translocated ^13^C			Translocated ^15^N		
		Sum Sq^a^	*F*	*P* ^b^	Sum Sq^a^	*F*	*P* ^b^
Direction	1	**7.37**	**10.17**	**0.002**	**87.03**	**44.12**	**<0.001**
M shaded	1	**11.15**	**15.38**	**<0.001**	**21.45**	**10.87**	**0.002**
D shaded	1	0.09	0.12	0.733	6.35	3.22	0.077
Time	2	2.13	1.47	0.236	**19.34**	**4.90**	**0.010**
Direction: M shaded	1	0.47	0.64	0.425	5.67	2.88	0.094
Direction: D shaded	1	0.03	0.04	0.841	4.13	2.09	0.152
M shaded: D shaded	1	0.14	0.19	0.664	0.00	0.00	0.964
Direction: time	2	**43.38**	**29.92**	**<0.001**	**134.30**	**34.04**	**<0.001**
M shaded: time	2	4.36	3.01	0.056	7.35	1.86	0.163
D shaded: time	2	3.33	2.29	0.108	0.03	0.01	0.993
Direction: M shaded: D shaded	1	0.01	0.02	0.897	2.29	1.16	0.285
Direction: M shaded: time	2	**13.22**	**9.12**	**<0.001**	8.82	2.24	0.114
Direction: D shaded: time	2	2.58	1.78	0.177	11.73	2.97	0.057
M shaded: D shaded: time	2	3.64	2.51	0.088	**12.45**	**3.16**	**0.049**
Direction: M shaded: D shaded: time	2	0.46	0.32	0.730	0.81	0.21	0.815
Residuals	72	52.20			142.04		

^a^Sum of squares type I was used.

^b^Effects with *P*<0.05 are highlighted in bold.

**Fig. 2.**
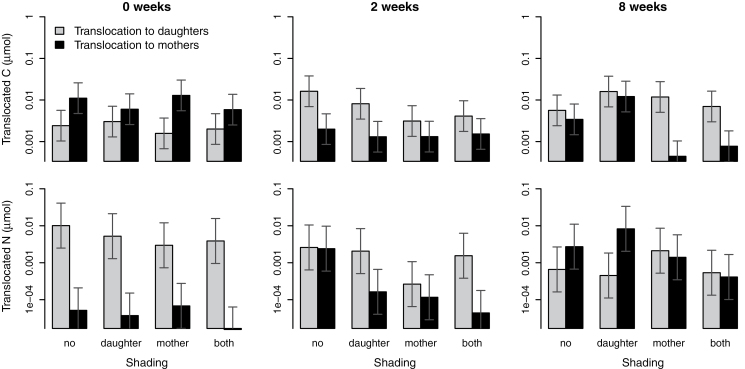
Amounts of translocated ^13^C and ^15^N (means and 95% confidence intervals) from labelled mothers towards daughter ramets and from labelled daughters towards mother ramets under different shading treatments and at different times from the establishment of daughter ramets. Note that the *y*-axes are log scales.

**Fig. 3.**
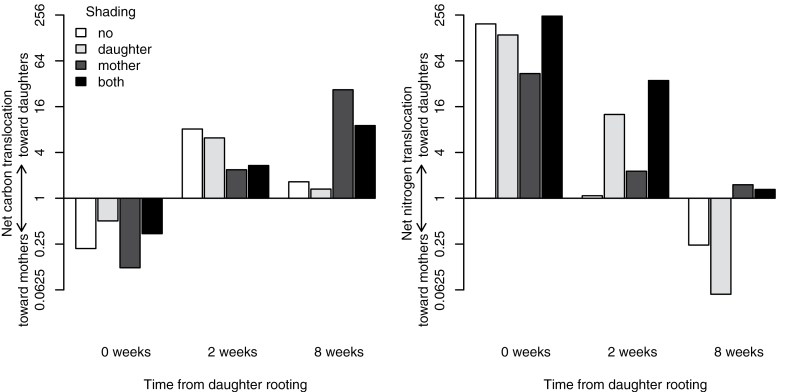
Net carbon and nitrogen translocation between mother and daughter ramets expressed as the log ratio of translocated label towards daughters and towards mothers. Values above 1 indicate prevailing translocation towards daughters, whereas values below 1 indicate prevailing translocation towards mothers.

At the beginning of daughter establishment (first harvest), more carbon was translocated towards mothers than towards daughters, with no significant effect of shading (Fig. 2). Two weeks after daughter establishment, the translocation of carbon towards mothers declined, whereas the translocation towards daughters rose relative to the first harvest regardless of the light treatment (Fig. 2), resulting in net flow of carbon directed towards daughters (Fig. 3). Shading had no significant effect on carbon translocation at the second harvest. Eight weeks after daughter establishment, carbon translocation towards unshaded mothers was lower than at the first harvest, resulting in balanced carbon translocation in both directions (Fig. 2). However, when mothers were shaded, translocation towards mothers declined considerably, and net carbon flow was consequently directed towards daughters (Figs 2, 3).

The fraction of assimilated ^13^C exported towards unlabelled ramets significantly decreased with time. Daughters initially exported proportionally more labelled carbon than mothers, but this difference was reversed and eventually diminished at the second and the final harvests, respectively. Furthermore, daughters initially exported a smaller proportion of the ^13^C when shaded, whereas shading had no effect on ^13^C export from daughters at the second harvest, and shaded daughters exported a higher proportion of the label at the final harvest. At the time of the final harvest, daughters exported a markedly smaller proportion of labelled carbon towards shaded mothers than when the mothers were unshaded (Tables 3, 4).

**Table 3.** Fractions of assimilated ^13^C and ^15^N exported from labelled ramets towards unlabelled ramets

	Exported carbon						Exported nitrogen					
	From mothers			From daughters			From mothers			From daughters		
	Fitted (%)^a^	95% CI^b^		Fitted (%)	95% CI		Fitted (%)	95% CI		Fitted (%)	95% CI	
**First harvest**												
Full light	2.70	1.01	7.19	19.27	7.23	51.32	35.34	9.59	130.30	25.26	6.85	93.14
Daughter shaded	3.42	1.28	9.10	7.53	2.83	20.05	26.61	7.22	98.11	15.50	4.20	57.13
Mother shaded	2.55	0.96	6.79	12.65	4.75	33.70	30.02	8.14	110.65	14.03	3.80	51.71
Full shade	2.89	1.09	7.70	3.84	1.44	10.22	42.80	11.61	157.77	13.77	3.73	50.75
**Second harvest**												
Full light	2.91	1.09	7.76	0.78	0.29	2.08	2.39	0.65	8.80	6.56	1.78	24.18
Daughter shaded	2.31	0.87	6.16	0.55	0.21	1.47	3.25	0.88	11.99	2.91	0.79	10.73
Mother shaded	2.18	0.82	5.82	0.67	0.25	1.80	2.62	0.71	9.65	0.76	0.21	2.81
Full shade	2.04	0.76	5.42	1.30	0.49	3.46	8.54	2.32	31.47	2.43	0.66	8.97
**Final harvest**												
Full light	0.54	0.20	1.44	0.39	0.15	1.04	0.31	0.08	1.13	1.42	0.39	5.24
Daughter shaded	1.23	0.46	3.27	1.90	0.71	5.06	0.20	0.06	0.75	14.44	3.92	53.23
Mother shaded	2.24	0.84	5.98	0.04	0.02	0.11	3.36	0.91	12.38	0.59	0.16	2.16
Full shade	4.20	1.58	11.18	0.38	0.14	1.02	3.22	0.87	11.86	1.36	0.37	5.03

^a^Expected (untransformed) values of the variable in the model (using log-transformed values).

^b^Limits of 95% confidence intervals (CI).

**Table 4.** ANOVA of linear models of the percentage of exported ^13^C and ^15^N (log-transformed) from labelled ramets in response to direction of translocation, shading of mothers (M) and daughters (D), and time of labelling

	d.f.	Exported ^13^C			Exported ^15^N		
		Sum Sq^a^	*F*	*P* ^b^	Sum Sq^1^	*F*	*P* ^b^
Direction	1	**6.04**	**6.25**	**0.015**	0.23	0.13	0.717
M shaded	1	0.84	0.87	0.355	0.01	0.01	0.941
D shaded	1	2.05	2.12	0.149	2.83	1.65	0.203
Time	2	**64.24**	**33.25**	**<0.001**	**139.00**	**40.56**	**<0.001**
Direction: M shaded	1	**6.54**	**6.77**	**0.011**	**27.33**	**15.95**	**<0.001**
Direction: D shaded	1	0.04	0.04	0.838	0.59	0.35	0.559
M shaded: D shaded	1	0.27	0.28	0.597	1.34	0.78	0.379
Direction: time	2	**34.05**	**17.62**	**<0.001**	9.97	2.91	0.061
M shaded: time	2	0.81	0.42	0.660	2.70	0.79	0.459
D shaded: time	2	**13.42**	**6.94**	**0.002**	2.65	0.77	0.466
Direction: M shaded: D shaded	1	0.41	0.42	0.519	0.14	0.08	0.779
Direction: M shaded: time	2	**15.70**	**8.12**	**0.001**	**14.21**	**4.15**	**0.020**
Direction: D shaded: time	2	**6.08**	**3.15**	**0.049**	6.74	1.97	0.147
M shaded: D shaded: time	2	0.58	0.30	0.740	3.93	1.15	0.323
Direction: M shaded: D shaded: time	2	0.33	0.17	0.843	2.16	0.63	0.535
Residuals	72	69.57			123.37		

^a^Sum of squares type I was used.

^b^Effects with *P*<0.05 are highlighted in bold.

### Translocation of nitrogen

The amount of translocated nitrogen was significantly affected by the direction of translocation, the time from the establishment of daughter ramets, and the shading of mother ramets, of which the shading generally decreased nitrogen translocation. The effect of the direction changed significantly with time (see Table 2).

At the beginning of daughter establishment, the amount of nitrogen translocated towards daughters was higher than that of nitrogen translocated towards mothers, with the light treatment having no effect (Fig. 2). Two weeks after daughter establishment, translocation towards mothers rose to values roughly equivalent to translocation towards daughters (Fig. 2). Absolute translocation towards daughters did not significantly differ from that observed at the first harvest. There was no significant effect of shading on nitrogen translocation at the second harvest. Eight weeks after daughter establishment, nitrogen translocation towards mothers increased, whereas translocation towards daughters declined relative to the first harvest, resulting in slightly higher net translocation of nitrogen towards mothers than towards daughters (Figs 2, 3).

The fraction of assimilated ^15^N exported towards unlabelled ramets significantly decreased with time. Daughters exported a smaller proportion of labelled nitrogen towards shaded mothers, especially at the time of the final harvest. Correspondingly, shaded mothers exported proportionally more labelled nitrogen towards daughters at the time of the final harvest (Tables 3, 4).

### Effect of integration on ramet growth

**Fig. 4.**
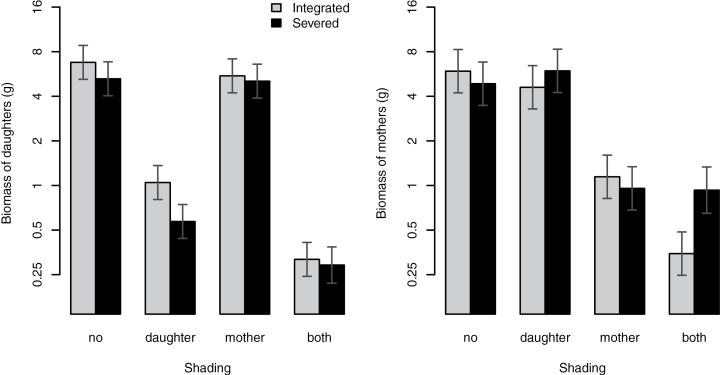
Final dry biomass (log scale, means and 95% confidence intervals) of daughter and mother ramets with intact or severed connections under different shading treatments.

Shading had a significant effect on the growth of both daughter and mother ramets. In daughter ramets, integration had a positive effect on growth, with no significant interactions of integration and shading. In mother ramets, the effect of integration alone was not significant, but integration with shaded daughters had a marginally significant negative effect on the growth of mothers (Table 5, Fig. 4)

**Table 5.** ANOVA of linear models of final biomass of daughter and mother ramets (log-transformed) in response to shading of mothers (M), shading of daughters (D), and integration

	d.f.	Daughter ramet biomass			Mother ramet biomass		
		Sum Sq^a^	*F*	*P* ^b^	Sum Sq^a^	*F*	*P* ^b^
Initial size	1	**0.58**	**4.53**	**0.038**	**14.87**	**95.34**	**<0.001**
M shaded	1	**3.56**	**27.99**	**<0.001**	**51.41**	**329.53**	**<0.001**
D shaded	1	**93.51**	**735.7**	**<0.001**	**1.13**	**7.26**	**0.009**
Integration	1	**1.24**	**9.77**	**0.003**	0.51	3.24	0.078
M shaded: D shaded	1	**2.74**	**21.54**	**<0.001**	**0.79**	**5.04**	**0.029**
M shaded: integration	1	0.43	3.39	0.071	0.44	2.83	0.098
D shaded: integration	1	0.39	3.07	0.085	**0.64**	**4.1**	**0.048**
M shaded: D shaded: integration	1	0.11	0.89	0.349	0.41	2.61	0.112
Residuals	54	6.86			8.43		

^a^Sum of squares type I was used.

^b^Effects with *P*<0.05 are highlighted in bold.

## Discussion

Our results showed changes in the translocation of both above-ground (carbon) and below-ground (nitrogen) resources between mother and daughter ramets of *Agrostis stolonifera* during the course of daughter ramet development, as well as an effect of light gradients on resource translocation. Resource translocation in the initial stages of daughter ramet development (ontogeny) was generally in accordance with our expectation of initial resource translocation directed towards daughters. Nitrogen was translocated predominantly towards daughters at the very beginning of their establishment, and carbon translocation was directed towards the rapidly developing daughters 2 weeks after the beginning of their rooting (Fig. 3). Contrary to our predictions, absolute translocation of resources during early daughter ramet development was not affected by light availability to the different ramets. Overall, the magnitude of absolute resource flows did not decline with time as we originally expected, although individual ramets were obviously self-sustaining in terms of resource acquisition at the late developmental stage, i.e. 8 weeks after daughter rooting (Fig. 2). The amounts exported from labelled ramets, however, accounted for smaller proportions of the total assimilated resources at later stages of development than at the beginning (Table 3). Light conditions significantly altered carbon translocation only at the late developmental stage (i.e. 8 weeks after daughter rooting) and, unexpectedly, the net translocation of carbon (Fig. 3) as well as the proportional export of assimilated nitrogen (Table 3) were directed from shaded mothers towards daughters, irrespective of daughter light conditions.

Photosynthates are translocated by the phloem, and their movement in the plant body is determined primarily by the activity of sinks and sources. Nitrogen as well as other phloem-mobile nutrients can be translocated by both the transpiration flow in the xylem and by the phloem (Marshall, 1990). Transport of resources thus has a rather complex nature, as plants can, for example, further modify the phloem flow by hormonal control (Alpert *et al.*, 2002).

### Resource translocation during ramet development

Translocation of resources to emerging ramets as well as the crucial role of integration in early development of new ramets are well documented in the literature (e.g. Hartnett and Bazzaz, 1983; Alpert, 1996; Xu *et al.*, 2012), but it is not clear if resource gradients alter maternal support at this stage (Alpert and Mooney, 1986). We observed that carbon was translocated predominantly from establishing daughters towards mothers at the very beginning of daughter rooting (Fig. 3), which showed that the developed tillers that formed daughter ramets were self-reliant in terms of carbon assimilation and, at the same time, smaller tillers continuously being formed on the mother ramet functioned as a strong sink for carbon. At that time, the resource exchange between ramets probably still corresponded to the resource exchange among unrooted tillers of a single ramet, which function essentially as leaves. Initial carbon translocation may, however, be directed towards daughter ramets in other plant species with different ramet morphology (e.g. daughter ramets having smaller leaf area in comparison to mothers). For example, translocation of carbon from older leaves to unrooted daughter nodes was observed in white clover (Kemball and Marshall, 1995). Net carbon translocation in our study was not significantly affected by light gradients at the initial stage, although shaded daughters exported proportionally less carbon towards mothers (Table 3). In contrast, Alpert and Mooney (1986) reported that carbon translocation towards unrooted ramets of *Fragaria chiloensis* was induced by shading these ramets. The observed initial strong nitrogen translocation towards almost-unrooted daughters was inevitable, even though uptake of labelled nitrogen applied to daughters and its translocation towards mothers were measurable (evidencing uptake of nitrogen by very small roots of the daughters, Fig. 2).

At the time of the initial rapid daughter growth (2 weeks after the beginning of rooting), carbon translocation was directed towards daughters, with the shading treatment having no significant effect (Fig. 3). The developing daughter roots and emerging daughter tillers thus probably formed the main sink for shared carbon reserves, and relative sink strength was not markedly affected by shading. Nitrogen translocation was balanced in both directions, with the shading treatment having no significant effect, although slower-growing shaded daughters seemingly still gained nitrogen support from mothers (Fig. 3). Total absolute nitrogen flow did not decline as we expected (Fig. 2). Daughter ramets therefore gained independence in terms of nitrogen uptake relatively soon after establishment, although the bidirectional flow between ramets had not ceased. In comparison, translocation of nitrogen was directed mainly towards the younger ramets in clonal fragments of *Fragaria chiloensis*, with only little reverse translocation (Alpert, 1996). However, bidirectional translocation, and thus net nutrient flow, has generally not been examined, which complicates comparisons of other studies with our results.

When daughters reached the size of mothers and ramet size was determined only by the shading treatment (8 weeks after rooting, Table 1), resource translocation patterns in *A. stolonifera* surprisingly seemed to be altered only by shading of mothers (Table 2). Carbon exchange between unshaded mothers and their developed daughters was balanced irrespective of daughter light conditions, while it was directed from shaded mothers towards daughters, which restricted export to light-limited mothers (Figs 2, 3). In contrast, net translocation of nitrogen was directed from developed daughters, whether shaded or not, towards mothers (Fig. 3). Although the effect of shading alone was not statistically significant for nitrogen translocation, the translocation towards unshaded mothers seems to be the main contributor to this directionality (Fig. 3). At the same time, light-limited mothers enhanced their proportional export of both resources towards daughters, and the reverse proportional export from daughters declined (Table 3). Similarly, the export of nitrogen towards unshaded ramets was enhanced by shading of labelled ramets in *Sasa palmata* (Saitoh *et al.*, 2006). Our results contrast with predicted higher translocation of photosynthates towards shaded ramets and only partly support the prediction of nitrogen translocation towards faster-growing, unshaded ramets at the late developmental stage (i.e. 8 weeks after rooting). On the other hand, translocation among established ramets has been shown to be enhanced by environmental resource gradients in several species (Marshall, 1990; Saitoh *et al.*, 2006), and the ability to support resource-limited ramets is considered one of the main advantages of clonal integration (Song *et al.*, 2013). For example, carbon translocation from mothers to 8-week-old daughters was enhanced by shading of daughters in *Alternanthera philoxeroides* (Xu *et al.*, 2010), and severe shading of mother ramets induced translocation of carbon from established daughters back to the mothers in *Lathyrus sylvestris* (Magda *et al.*, 1988).

The translocation patterns that we observed indicate that the spread of clonal fragments of *A. stolonifera* to new sites is preferred to the maintenance of growth of developmentally older ramets in light-limiting conditions. On the other hand, when originally growing at a favourable patch, clonal fragments did not reallocate resources to already established daughter ramets and, instead, mother ramets seemed to use nitrogen from newly occupied daughter patches to support their own growth. Younger ramets may therefore serve as a supplementary source of mineral nutrients for older ramets, as was also observed in *Populus tremuloides* (Pinno and Wilson, 2014), although the mother ramets were much larger than daughters in that case.

Contrary to our expectations, the magnitude of absolute carbon and nitrogen flows between ramets did not change significantly during daughter ramet development (although the proportions of exported labels declined, and net translocation markedly changed during development), and ramets thus remained physiologically interconnected, not only under heterogeneous conditions but also in homogeneous light conditions (Fig. 2). Consequently, translocation patterns in *A. stolonifera* are likely to respond readily to a change of local conditions or to stress. The maintained bidirectional nitrogen flow may partly be caused by different sites of energetically demanding reduction of nitrogen and its subsequent utilization (Li and Wang, 2011).

Our results also illustrate the necessity of bidirectional tracing of resource translocation to estimate net flows of resources in clonal plant systems. Although the translocation detected in one direction could be high, we have shown that it can be accompanied by an equally high reverse translocation, resulting in a near-zero net flow between ramets. To date, however, only a few studies have examined bidirectional translocation of labelled resources between ramets (Alpert *et al.*, 1991; Alpert, 1996; Pinno and Wilson, 2014).

### Effect of integration on ramet growth

The positive effect of integration on the growth of daughter ramets (Fig. 4) probably reflects maternal support with both carbon and nitrogen at the initial stages of daughter development. The integration effect on daughter growth was not significantly modified by shading treatment, despite the observed effect of shading on the pattern of translocation. However, shading affected the translocation pattern only in the late stage of ramet development, which may have been too late to be detectable in terms of differences in biomass at the time of harvest. In mothers, there was a marginally significant indication of the cost of integration when daughters were shaded. However, this effect did not have a clear relationship with the observed translocation patterns of carbon and/or nitrogen between the ramets.

Similar to our results, shading of daughters affected neither the amount of translocated carbon nor the effect of integration on growth in *Phyla canescens* (Xu *et al.*, 2010). In contrast, both carbon import and the effect of integration on growth were higher in shaded daughter ramets of *Alternanthera philoxeroides* (Xu *et al.*, 2010, 2012). Only a few studies, however, have combined tracing of labelled elements with analyses of the effect of integration on growth (see D’Hertefeldt and Jonsdottir, 1994; Alpert, 1996; Saitoh *et al.*, 2006; Xu *et al.*, 2010), so it is not currently clear to what extent the resource translocation at different developmental stages is reflected in ramet growth.

### Role of integration in competition for light

Enhanced benefits of integration under competitive conditions have been demonstrated for only a few plant species (Saitoh *et al.*, 2002; Roiloa *et al.*, 2010; Xu *et al.*, 2012; Wang *et al.*, 2016). In other studies, including ours, the overall benefits of integration on growth were not significantly altered by competition (e.g. Březina *et al.*, 2006; Yu *et al.*, 2009; Glover *et al.*, 2015), or there were even lower benefits of integration with versus without (above-ground) competition (Pennings and Callaway, 2000). We therefore suggest that in some species, integration may enhance performance of ramets under competition through their receipt of preferential support (Xu *et al.*, 2010). On the other hand, in other species, including *Agrostis stolonifera*, resources may be translocated among ramets to maximize efficient space exploration and exploitation of favourable patches. Nevertheless, it is not clear to what extent the resource-sharing strategy varies among species. It is conceivable that it may differ among species with different ecological strategies or among species from different environments, and therefore the current results should only be generalized with caution.

## Supplementary data

Supplementary data are available at *JXB* online.

Fig. S1. Variations in temperature and light intensity under full-light and shade treatments during the course of the experiment.

Fig. S2. Equipment used for labelling with ^13^CO_2_.

Fig. S3. Example of a ramet pair before the final harvest.

**Data deposition**

Biomass and translocation data are available at Dryad Digital Repository. https://doi.org/10.5061/dryad.f6q5j

Supplementary Data
