# Supplementary material for: The direction of carbon and nitrogen fluxes between ramets in Agrostis stolonifera changes during ontogeny under simulated competition for light
Source: J Exp Bot. 2018 Feb 21;69(8):2149–58. doi: 10.1093/jxb/ery068 (PMC6020861; doi:10.1093/jxb/ery068)
Supplement: Supplementary Data [file ery068_suppl_supplementary_data.pdf]

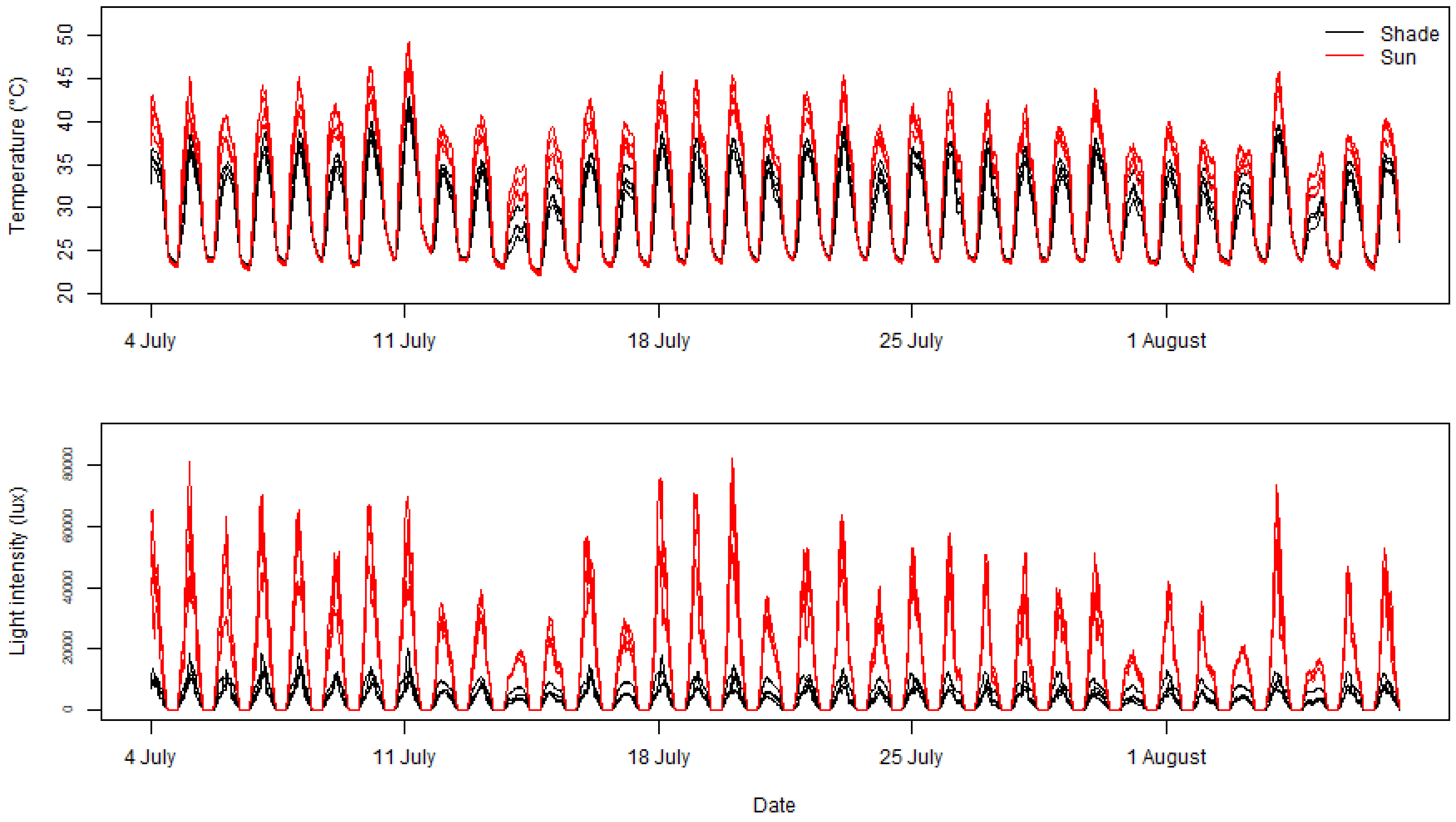

Figure S1. Course of temperature and light intensity under full light (red lines) and shade (black lines) treatments.

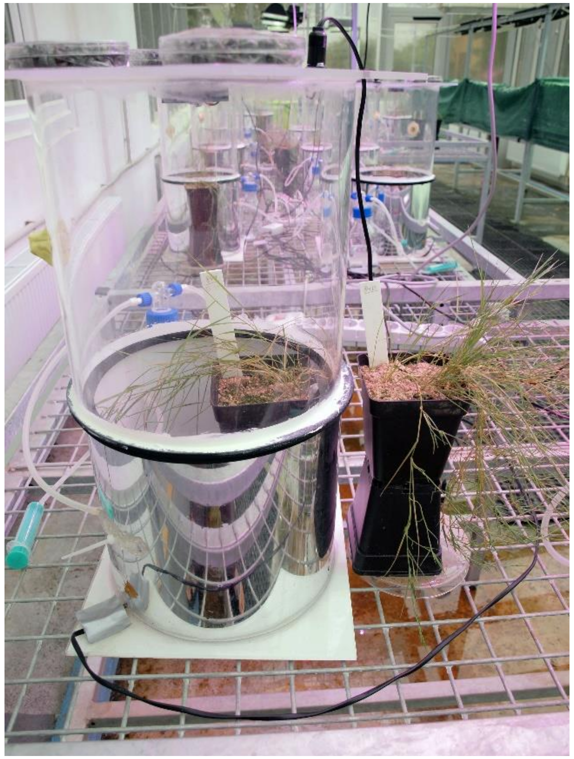

Figure S2. Labelling by $^{13}CO_2$. A labelled ramet (left) was enclosed in a plexiglass chamber and $^{13}CO_2$ was released inside the chamber.

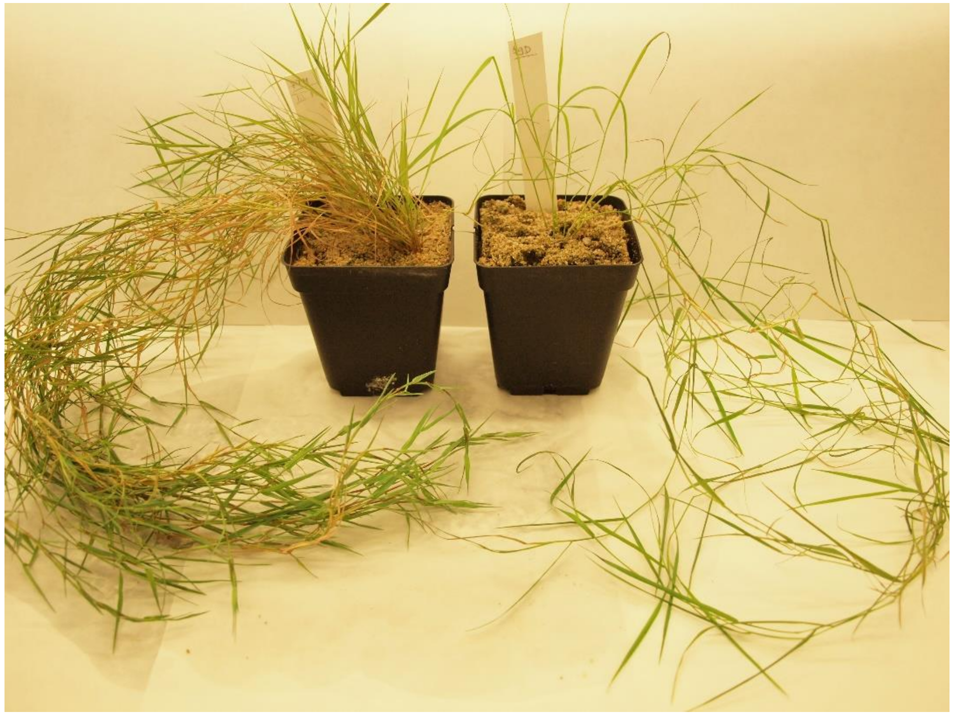

Figure S3. Integrated ramet pair before final harvest. The ramets grown in the sun (left) had on average 4.4–4.5 times higher biomass than the shaded ramets (right).
